# Adoption and Efficiency of an Anesthesia Information Management System: Evaluation of Workflow Integration in Perioperative Care

**DOI:** 10.3390/healthcare14111520

**Published:** 2026-05-30

**Authors:** Nikola Prpic, Ileana Lulic, Laura Karla Bozic, Mario Staresinic, Dinka Lulic, Dinko Tonkovic, Neven Henigsberg, Iva Bacak Kocman, Gorjana Erceg, Jadranka Pavicic Saric

**Affiliations:** 1 Department of Anesthesiology, Intensive Care and Pain Medicine, Clinical Hospital Merkur, Zajceva 19, 10 000 Zagreb, Croatia; nikolaprpic2@gmail.com (N.P.); blaura210@gmail.com (L.K.B.); bacakkocmaniva@gmail.com (I.B.K.); gorjanaerceg@gmail.com (G.E.); jadranka.pavicic.saric@kb-merkur.hr (J.P.S.); 2 Department of Medical Biochemistry and Hematology, Faculty of Pharmacy and Biochemistry, A. Kovacica 1, 10 000 Zagreb, Croatia; dinka.lulic@gmail.com; 3 Department of Surgery, Clinical Hospital Merkur, Zajceva 19, 10 000 Zagreb, Croatia; mario.staresinic@kb-merkur.hr; 4 Immediate Medical Care Unit, Saint James Hospital, SLM-1030 Sliema, Malta; 5 Department of Anesthesiology, Reanimatology, Intensive Care and Pain Medicine, Clinical Hospital Center Zagreb, Kispaticeva 12, 10 000 Zagreb, Croatia; dtonkovi@mef.hr; 6 Department of General and Forensic Psychiatry and Clinical Psychophysiology, Psychiatric Hospital Vrapce, Bolnicka cesta 32, 10 090 Zagreb, Croatia; neven@henigsberg.me

**Keywords:** anesthesia information management systems, perioperative care, clinical decision support systems, electronic health records, workflow integration

## Abstract

**Background:** Anesthesia Information Management Systems (AIMSs) support perioperative documentation and clinical decision-making, but their real-world adoption remains heterogeneous and incompletely understood. **Methods:** This study combined a cross-sectional survey with a randomized crossover simulation study conducted at a tertiary care center following AIMS implementation. All anesthesiologists were invited to complete a structured questionnaire assessing satisfaction, usability, adoption, and use of decision-support functionalities. In the simulation study, participants entered standardized intraoperative data into both paper-based records and the electronic AIMS, with documentation time recorded. Survey data were analyzed descriptively with subgroup analyses, and documentation times were compared using the Wilcoxon signed-rank test. **Results:** A total of 27 anesthesiologists participated. Overall satisfaction and workflow integration were high, with 81.48% reporting that the system was easy to use and well-integrated into clinical practice. Electronic documentation was preferred across multiple domains, including time efficiency (92.59%) and accuracy (85.19%). In the simulation study, electronic documentation was significantly faster than paper-based documentation (median 540 vs. 1140 s; *p* = 0.0016). Adoption patterns demonstrated a bimodal distribution, with no association with technological literacy or engagement with educational materials. Decision-support features embedded within routine workflows were used more frequently than those requiring additional navigation. **Conclusions:** AIMS implementation was associated with high user satisfaction and improved documentation efficiency, but showed heterogeneous adoption and selective feature use. Effective integration appears to depend on workflow alignment rather than user characteristics alone.

## 1. Introduction

Patient data management systems (PDMSs) are an integral component of modern healthcare, providing structured solutions for medical documentation and the organization and visualization of patient information across diverse clinical settings [1]. As healthcare systems continue to undergo digital transformation, such platforms increasingly serve not only as repositories of clinical data but also as tools that reduce administrative burden and improve clinical efficiency, which may contribute to reducing physician burnout [2].

Within perioperative medicine, the complexity and temporal density of anesthesia care have necessitated the development of specialized PDMSs, namely Anesthesia Information Management Systems (AIMSs). These systems extend beyond conventional documentation by enabling real-time integration of physiological data, automated recording of intraoperative events, and support for clinical decision-making, including drug dosing and event awareness [3]. In this context, AIMSs represent an important interface between digital systems and perioperative clinical practice.

**Figure 1:**
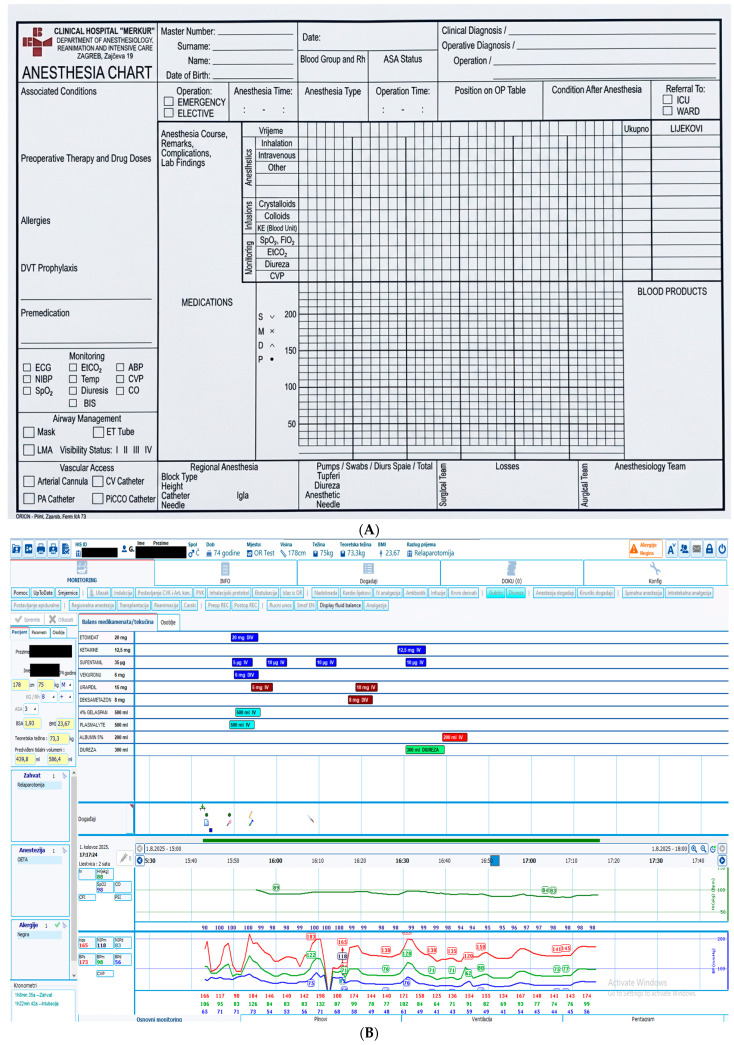
Comparison of anesthesia documentation formats used at the study center: (**A**) traditional paper-based anesthesia record and (**B**) electronic Anesthesia Information Management System (AIMS) interface.

The AIMS implemented at our institution was the Diane system (Bow Medical, France), introduced following a structured period of local configuration and integration to align with existing departmental practices. Compared with traditional paper-based anesthesia records, which are characterized by limited temporal resolution and reduced clarity during periods of intensive documentation (Figure 1A), electronic AIMS platforms enable structured, high-resolution data capture and improved accessibility of perioperative information (Figure 1B). These differences formed part of the conceptual basis for evaluating documentation efficiency, usability, and patterns of system adoption in the present study.

Despite these capabilities, the adoption of AIMSs in clinical practice has been associated with variable success [4,5,6]. Existing literature has predominantly focused on implementation strategies, system characteristics, and user satisfaction, with a substantial body of work describing deployment processes and adoption prevalence [7,8,9]. However, adoption and sustained routine use of these systems remain heterogeneous, and the factors that determine successful implementation in real-world perioperative environments are not fully understood.

Several determinants of technology adoption in healthcare have been proposed, including prior technical experience, user involvement in system design, and the availability of educational resources such as video-based training [10,11,12]. While these factors have been examined individually, their combined influence within a single implementation context, particularly in high-acuity perioperative settings, has not been well characterized. Furthermore, the relationship between system availability and the actual utilization of its functionalities in routine clinical practice remains unclear.

Therefore, the aim of this study was to characterize the implementation of an AIMS in a tertiary clinical setting and to evaluate factors associated with its adoption by integrating assessment of user perceptions with objective measurement of documentation performance.

## 2. Methods

### 2.1. Study Design and Setting

This study consisted of a cross-sectional survey and a randomized crossover simulation study. It was conducted at the Department of Anesthesiology, Intensive Medicine and Pain Medicine, Clinical Hospital Merkur, Zagreb, Croatia, following the implementation of an AIMS in 2025.

### 2.2. Participants

All anesthesiologists employed at the department at the time of the study were eligible for participation in the survey, representing the entire target population within the study setting. The survey link was distributed exclusively within the department to ensure that only eligible participants responded. Participation was voluntary, and an informed consent statement was provided at the beginning of the survey.

For the simulation study, anesthesiologists were recruited independently through an open call posted on the department notice board, and all applicants were included. Participants in the simulation study were not required to have participated in the survey component of the study.

Demographic variables collected included age, sex, and length of anesthesia practice.

### 2.3. Survey Design and Data Collection

The questionnaire was developed in consultation with statistical experts to assess user perceptions, usability, documentation practices, and decision-support functionalities related to AIMS implementation. Prior to distribution, the questionnaire underwent internal review to assess the clarity, relevance, and comprehensibility of survey items. Internal consistency reliability of the questionnaire was assessed using Cronbach’s alpha, which was 0.76, indicating acceptable reliability. Formal psychometric validation and factor analysis were not performed due to the exploratory nature of the study and the limited sample size associated with the single-center implementation setting.

It was administered using Google Forms and comprised 47 questions organized into five sections: demographic characteristics, overall satisfaction with the AIMS, comparison between paper-based and electronic documentation, ease of system use, and perceived decision-support capabilities. The questionnaire was intentionally structured to capture multiple dimensions of AIMS adoption and user interaction within the perioperative setting.

Responses were obtained over a two-week period. Survey participation was anonymous, and access to the dataset was restricted to members of the study team to maintain data confidentiality.

The complete list of survey questions and response options is provided in Table 1.

### 2.4. Simulation Study

To evaluate documentation workload, participants were provided with identical synthetic data representing a two-hour surgical procedure. The dataset included induction and maintenance medication, performed procedures (endotracheal intubation, central venous catheter placement, and arterial catheter placement), and physiological parameters.

Each participant entered the data into both paper-based records and the electronic AIMS. Participants were randomly assigned to one of two sequences: paper documentation followed by electronic documentation, or the reverse order. The crossover design was selected to minimize inter-individual variability in documentation speed and familiarity with anesthesia records by allowing each participant to serve as their own control.

The time required to complete each documentation task was recorded in seconds.

### 2.5. Outcomes

Primary outcomes were user satisfaction and adoption of the AIMS, assessed through survey responses.

Secondary outcomes included documentation time for paper-based and electronic systems, as well as patterns of use of AIMS decision-support functionalities.

### 2.6. Statistical Analysis

Survey data were summarized using frequency distributions and presented as counts and percentages.

For questions with heterogeneous response distributions, exploratory subgroup analyses were conducted based on variables such as age and technological literacy using chi-square tests or gamma coefficient analysis, as appropriate. Given the relatively small subgroup sizes, these analyses were interpreted cautiously.

In the simulation study, differences in documentation time between paper-based and electronic systems were analyzed using the Wilcoxon signed-rank test.

Statistical significance was defined as a two-sided *p*-value <0.05. All analyses were performed using Microsoft Excel.

### 2.7. Ethical Approval

The study was approved by the Ethics Committee of Clinical Hospital Merkur (UR-BR: 03/1-7035). Informed consent was obtained from all survey participants.

## 3. Results

### 3.1. Study Population

The survey was completed by 27 anesthesiologists out of 37 eligible participants (response rate: 73%). A complete distribution of response frequencies is provided in Table 2.

Among respondents, 22% were male and 78% female, with 67% attending physicians and 33% residents. The median age was 40 years (IQR 32.5–45.5), and the median duration of anesthesia practice was 10.8 years (IQR 4.5–14.5).

Self-reported technological literacy was high overall, with 4 respondents reporting very high literacy, 10 reporting high or moderate literacy, and 3 reporting low literacy; none reported absence of digital literacy.

### 3.2. User Satisfaction and System Performance

Responses indicated high overall satisfaction with the AIMS and favorable integration into clinical workflow.

A total of 81.48% of respondents agreed that the system was easy to use (59.26% completely, 22.22% partially), while 81.48% agreed that it fit well into their work (66.67% completely, 14.81% partially). Integration at the departmental level was supported by 55.56% of respondents who completely agreed.

Perceived benefits included accurate recording of intraoperative events and medications, as well as reduction of documentation errors.

**Figure 2:**
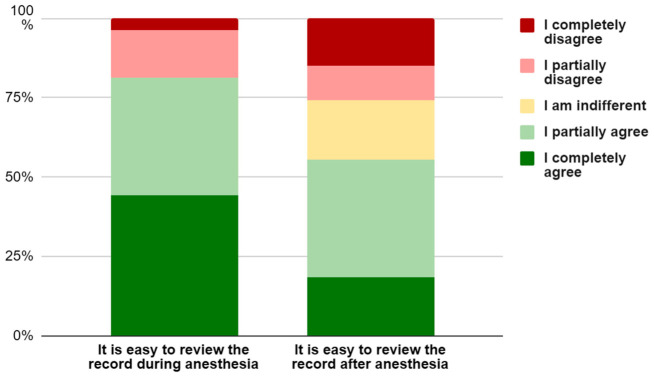
Reported ease of anesthesia record review during and after anesthesia.

A discrepancy was observed in record review, with higher agreement for intraoperative review (44.44% completely agree) compared with post-anesthesia review (18.52% completely agree) (Figure 2).

### 3.3. Comparison Between Paper-Based and Electronic Documentation

Electronic documentation was consistently preferred across multiple domains.

The AIMS was considered more time-efficient by 92.59% of respondents and more accurate in recording procedures by 85.19%. It was also associated with improved collaboration (59.26%), more accurate documentation of administered medications (81.48%), and increased time available for patient care (81.48%).

Long-term data storage was favored by 92.59% of respondents, and 88.89% reported an overall preference for electronic record keeping.

**Figure 3:**
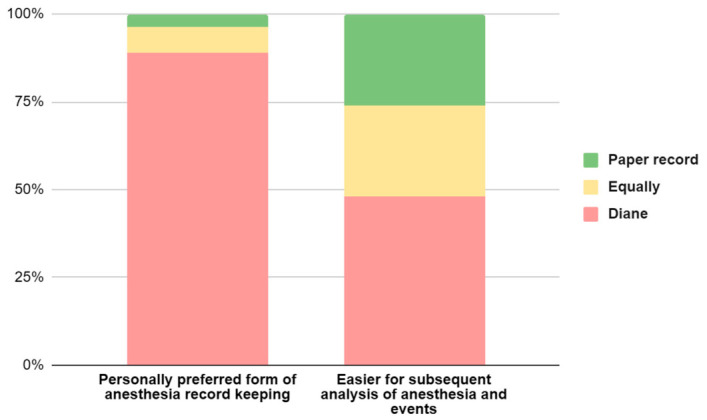
Comparison of personal preference and perceived ease of subsequent analysis of anesthesia records.

In contrast, responses regarding ease of subsequent analysis were more heterogeneous, with 48.15% favoring electronic records, 25.93% reporting no difference, and 25.93% favoring paper-based records (Figure 3).

### 3.4. Adoption Patterns and Training

**Figure 4:**
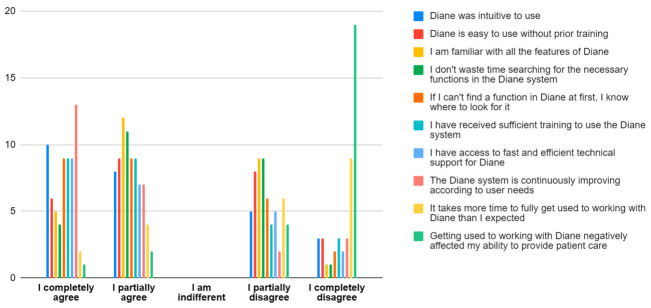
Distribution of responses to survey items assessing Anesthesia Information Management Systems (AIMSs) adoption.

Responses related to AIMS adoption demonstrated a bimodal distribution across multiple items (Figure 4).

In contrast to the bimodal response pattern observed across several adoption-related items, responses indicating that the system was intuitive to use (38.46% completely agree) and continuously improving according to user needs (52.00% completely agree) showed greater agreement. Similarly, most respondents indicated that adaptation to the system did not negatively affect patient care (73.08% completely disagree).

Regarding educational materials, 48.15% of respondents reported watching some videos, 33.33% reported no engagement, and 18.52% reported watching all available materials. Among those who engaged with the videos, perceived usefulness was high (median 4.0/5; IQR 3.75–5).

Exploratory subgroup analyses demonstrated no significant association between technological literacy and video engagement, nor between video use and perceived adequacy of training or system usability.

### 3.5. Utilization of Decision-Support Features Varied Across Functionalities

More frequent use was reported for features embedded within routine workflow, including guided record completion (51.85% reporting always or often), reduction of documentation errors (51.85%), and automated drug dosing calculations (70.37%).

**Figure 5:**
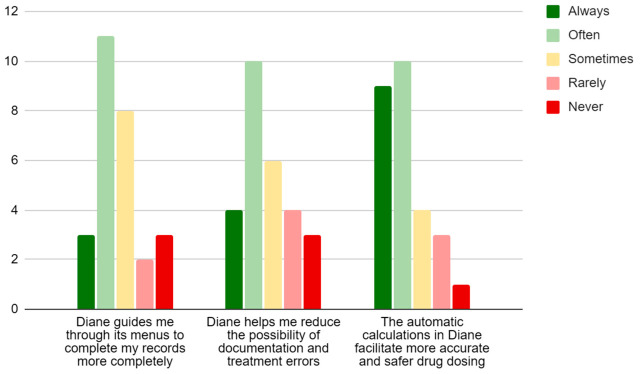
Distribution of responses to survey items assessing frequently used Anesthesia Information Management Systems (AIMSs) decision-support functions.

**Figure 6:**
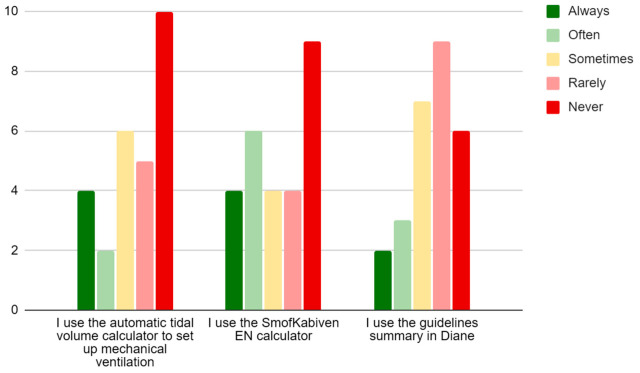
Distribution of responses to survey items assessing infrequently used Anesthesia Information Management Systems (AIMSs) decision-support functions.

Lower utilization was observed for specific tools requiring additional interaction, including the tidal volume calculator (37.04% reporting never use), SmofKabiven Extra Nitrogen calculator (33.33%), and guideline summary tools (22.22%) (Figure 5 and Figure 6).

Exploratory analyses demonstrated no significant correlation between the use of these functionalities and perceived system usability or workflow integration.

### 3.6. Simulation Study: Documentation Time

In the simulation study, 24 anesthesiologists completed documentation tasks using both paper-based records and the electronic AIMS. The median time required to complete electronic documentation was 540 s (IQR 480–600), compared with 1140 s (IQR 480–600) for paper-based documentation.

Electronic documentation was therefore 52.63% faster. This difference was statistically significant (Wilcoxon signed-rank test *p* = 0.001626; effect size 0.85).

## 4. Discussion

This study provides a comprehensive evaluation of AIMS implementation by integrating user perceptions with an objective assessment of documentation performance. The findings demonstrate high overall user satisfaction and a clear preference for electronic over paper-based documentation, accompanied by improved documentation efficiency. At the same time, adoption was heterogeneous, with a bimodal distribution of responses, and utilization of decision-support functionalities differed substantially across individual system features.

High levels of user satisfaction suggest successful incorporation of the AIMS into routine perioperative practice. These findings may reflect, at least in part, the structured implementation process, which included active involvement of end users during system configuration. Such user-centered approaches have previously been associated with improved acceptance of healthcare technologies [13,14]. Similarly, Jokar et al. highlighted the importance of perceived ease of use and user acceptance when evaluating an anesthesia electronic medical record system in the perioperative setting [15].

The observed satisfaction is likely multifactorial and may also be related to intrinsic advantages of electronic documentation systems, including improved temporal resolution, structured data capture, and enhanced clarity during periods of high clinical activity [16]. In contrast to paper-based records, electronic systems allow simultaneous documentation of multiple events without loss of interpretability, which may contribute to improved usability and perceived safety [16,17]. Nevertheless, the lower perceived ease of post-anesthesia record review compared with intraoperative review suggests that certain aspects of system usability and information retrieval may still require optimization. Comparable findings were reported by Quinzio et al., who observed that anesthesiologists using an AIMS in routine clinical practice considered the system to improve work quality and expressed a preference for electronic over paper-based documentation [4].

Nevertheless, the strong preference for electronic documentation observed in this study should be interpreted cautiously, as participant perceptions may have been influenced in part by recency effects associated with recent system implementation.

The results of the simulation study support these perceptions, demonstrating a significant reduction in documentation time with the electronic system. Although the simulation was conducted under controlled rather than real-world conditions, the findings provide objective evidence that electronic documentation may reduce administrative workload [18,19]. Given that documentation burden has been identified as a contributor to physician burnout, these improvements in efficiency may have relevant implications for perioperative practice [19,20]. Similarly, Kristobak et al. reported that embedding quality-assurance reporting within an existing AIMS substantially increased reporting rates, with reduced administrative burden considered a likely contributor to improved uptake [21]. However, the present study was not designed to assess clinical outcomes, and therefore any potential impact on patient safety or provider well-being should be interpreted cautiously.

A notable finding of this study is the presence of a bimodal distribution in responses related to AIMS adoption. This pattern suggests the coexistence of distinct user groups with differing levels of acceptance and system engagement despite uniform exposure to the same platform, highlighting the potential influence of additional unmeasured factors that may be better explored through qualitative approaches such as structured interviews or focus-group analyses. Neither self-reported technological literacy nor engagement with educational materials was associated with adoption patterns, indicating that commonly proposed predictors of technology uptake may not fully explain variability in high-acuity clinical settings [22,23]. These findings further suggest that additional, unmeasured factors, such as individual cognitive approaches, resistance to change, or perceived relevance of specific system features, may influence adoption behavior and warrant further investigation [24,25].

The analysis of decision-support functionalities revealed differential patterns of use, with higher utilization of features embedded within routine documentation processes and lower use of tools requiring additional navigation. These findings may suggest that close alignment with established clinical routines is an important determinant of feature utilization [26]. Functions integrated within the primary documentation interface may therefore be perceived as extensions of routine tasks, whereas tools requiring additional interaction may be used less frequently despite their potential clinical value [27,28]. However, the study did not include a qualitative assessment of user behavior or decision-making processes, and therefore, explanations for selective feature utilization should be interpreted cautiously.

The relationship between training and system use was also complex. Although educational video materials were rated as useful by participants who engaged with them, a substantial proportion of users did not utilize these resources, and no association was observed between video engagement and perceived system usability or adequacy of training. These findings may indicate a discrepancy between availability and uptake of educational interventions, suggesting that passive training formats alone may be insufficient to support comprehensive system adoption [29]. Structured or mandatory educational approaches, as well as incorporation of training into routine clinical activities, may therefore represent more effective implementation strategies [22,30].

Several limitations should be considered when interpreting these findings. First, this was a single-center study conducted within a specific institutional and organizational context, which may limit generalizability to other perioperative environments. The sample size was relatively small, particularly with respect to subgroup analyses, and the predominance of female respondents may have influenced perception-based findings. Survey responses were based on self-report and are therefore subject to response and social desirability bias. Furthermore, participation in the simulation study was voluntary, introducing the possibility of self-selection bias, as individuals with a greater interest in digital systems or higher technological confidence may have been more likely to participate.

Additional limitations relate to the implementation and simulation design itself. Involvement of members of the study team in the AIMS implementation process may have influenced both system configuration and participant perceptions. Moreover, although the simulation study enabled standardized comparison of documentation performance, it was conducted under controlled conditions and therefore does not fully replicate the complexity and time pressures of routine clinical practice. Future time–motion studies performed in live perioperative environments may provide a more accurate assessment of real-world workflow efficiency. Finally, the cross-sectional study design precludes assessment of longitudinal changes in adoption patterns and long-term system use.

## 5. Conclusions

The findings of this study indicate that successful AIMS implementation may require more than digitalization alone. User perceptions and simulation-based findings highlight the potential importance of structured onboarding, continuous user support, and integration of clinically relevant functionalities into everyday perioperative practice for sustained adoption and effective system use. Future implementation efforts should therefore focus not only on technical performance, but also on practical usability and long-term clinician engagement.

## Figures and Tables

**Table 1:** Survey questionnaire and response options.

Section	Question	Response Options
Demographics	What year did you start your residency in anesthesiology, resuscitation, and intensive care medicine?	Open-ended
Demographics	What year were you born?	Open-ended
Demographics	What is your sex?	Male; Female
Demographics	Do you participate in the education of residents/students?	Yes; No
Demographics	What is your position in the department?	Attending; Resident
Demographics	How would you rate your digital literacy?	Very high; High; Moderate; Low; None
AIMS satisfaction	Diane is easy to use.	I completely agree; I partially agree; I am indifferent; I partially disagree; I completely disagree
AIMS satisfaction	Diane fits well into my work.	I completely agree; I partially agree; I am indifferent; I partially disagree; I completely disagree
AIMS satisfaction	Diane has fit well into the work of the department.	I completely agree; I partially agree; I am indifferent; I partially disagree; I completely disagree
AIMS satisfaction	Diane satisfactorily records intraoperative events.	I completely agree; I partially agree; I am indifferent; I partially disagree; I completely disagree
AIMS satisfaction	Diane satisfactorily records administered medications.	I completely agree; I partially agree; I am indifferent; I partially disagree; I completely disagree
AIMS satisfaction	Diane allows me to monitor anesthesia more closely.	I completely agree; I partially agree; I am indifferent; I partially disagree; I completely disagree
AIMS satisfaction	It is easy to review the record during anesthesia.	I completely agree; I partially agree; I am indifferent; I partially disagree; I completely disagree
AIMS satisfaction	It is easy to review the record after anesthesia.	I completely agree; I partially agree; I am indifferent; I partially disagree; I completely disagree
AIMS satisfaction	Overall, I am satisfied with the Diane system.	I completely agree; I partially agree; I am indifferent; I partially disagree; I completely disagree
AIMS satisfaction	Thanks to Diane, I make fewer errors in documentation.	I completely agree; I partially agree; I am indifferent; I partially disagree; I completely disagree
AIMS satisfaction	Diane contributes to safer anesthesia management.	I completely agree; I partially agree; I am indifferent; I partially disagree; I completely disagree
Comparison	More time-efficient	Diane; Equally; Paper record
Comparison	Fits better into the work of anesthesiologists	Diane; Equally; Paper record
Comparison	More accurately records performed procedures	Diane; Equally; Paper record
Comparison	Enables better collaboration within the anesthesia team	Diane; Equally; Paper record
Comparison	Records administered medications more accurately	Diane; Equally; Paper record
Comparison	Provides more time to work on the patient	Diane; Equally; Paper record
Comparison	Safer for the patient	Diane; Equally; Paper record
Comparison	Ensures better long-term storage and preservation of records	Diane; Equally; Paper record
Comparison	Personally preferred form of anesthesia record keeping	Diane; Equally; Paper record
Comparison	Easier for subsequent analysis of anesthesia and events	Diane; Equally; Paper record
Comparison	More flexible for future improvements and adjustments	Diane; Equally; Paper record
Usability	Diane was intuitive to use.	I completely agree; I partially agree; I am indifferent; I partially disagree; I completely disagree
Usability	Diane is easy to use without prior training.	I completely agree; I partially agree; I am indifferent; I partially disagree; I completely disagree
Usability	I am familiar with all the features of Diane.	I completely agree; I partially agree; I am indifferent; I partially disagree; I completely disagree
Usability	I do not waste time searching for the necessary functions in the Diane system.	I completely agree; I partially agree; I am indifferent; I partially disagree; I completely disagree
Usability	If I cannot find a function in Diane at first, I know where to look for it.	I completely agree; I partially agree; I am indifferent; I partially disagree; I completely disagree
Usability	I have received sufficient training to use the Diane system.	I completely agree; I partially agree; I am indifferent; I partially disagree; I completely disagree
Usability	I have access to fast and efficient technical support for Diane.	I completely agree; I partially agree; I am indifferent; I partially disagree; I completely disagree
Usability	The Diane system is continuously improving according to user needs.	I completely agree; I partially agree; I am indifferent; I partially disagree; I completely disagree
Usability	It takes more time to fully get used to working with Diane than I expected.	I completely agree; I partially agree; I am indifferent; I partially disagree; I completely disagree
Usability	Getting used to work with Diane negatively affected my ability to provide patient care.	I completely agree; I partially agree; I am indifferent; I partially disagree; I completely disagree
Education	Have you watched the educational videos for Diane?	Yes; Some; No
Education	If you have watched at least part of the videos, to what extent were they useful to you?	Not useful; Slightly useful; Moderately useful; Very useful; Extremely useful
Decision-support	Diane guides me through its menus to complete my records more completely.	Always; Often; Sometimes; Rarely; Never
Decision-support	Diane guides me through its menus to complete my records more completely.	Always; Often; Sometimes; Rarely; Never
Decision-support	Diane helps me reduce the possibility of documentation and treatment errors.	Always; Often; Sometimes; Rarely; Never
Decision-support	I use the automatic tidal volume calculator to set up mechanical ventilation.	Always; Often; Sometimes; Rarely; Never
Decision-support	I use the SmofKabiven Extra Nitrogen calculator.	Always; Often; Sometimes; Rarely; Never
Decision-support	I use the guidelines summary in Diane.	Always; Often; Sometimes; Rarely; Never
Decision-support	The automatic calculations in Diane facilitate more accurate and safer drug dosing.	Always; Often; Sometimes; Rarely; Never

**Table 2:** Results of the survey questionnaire.

	I Completely Agree	I Partially Agree	I Am Indifferent	I Partially Disagree	I Completely Disagree
Diane is easy to use	16 (59.26%)	6 (22.22%)	1 (3.70%)	0 (0.00%)	4 (14.81%)
Diane fits well into my work	18 (66.67%)	4 (14.81%)	1 (3.70%)	1 (3.70%)	3 (11.11%)
Diane has fit well into the work of the Department	15 (55.56%)	7 (25.93%)	1 (3.70%)	2 (7.41%)	2 (7.41%)
Diane satisfactorily records intraoperative events	13 (48.15%)	9 (33.33%)	1 (3.70%)	0 (0.00%)	4 (14.81%)
Diane satisfactorily records administered medications	7 (25.93%)	14 (51.85%)	1 (3.70%)	3 (11.11%)	2 (7.41%)
Diane allows me to monitor anesthesia more closely	9 (33.33%)	9 (33.33%)	5 (18.52%)	1 (3.70%)	3 (11.11%)
It is easy to review the record during anesthesia	12 (44.44%)	10 (37.04%)	0 (0.00%)	4 (14.81%)	1 (3.70%)
It is easy to review the record after anesthesia	5 (18.52%)	10 (37.04%)	5 (18.52%)	3 (11.11%)	4 (14.81%)
Overall, I am satisfied with the Diane system	10 (37.04%)	11 (40.74%)	2 (7.41%)	3 (11.11%)	1 (3.70%)
Thanks to Diane, I make fewer errors in documentation	12 (44.44%)	6 (22.22%)	3 (11.11%)	2 (7.41%)	4 (14.81%)
Diane contributes to safer anesthesia management	12 (44.44%)	6 (22.22%)	4 (14.81%)	2 (7.41%)	3 (11.11%)
	Diane	Equally	Paper record		
More time-efficient	25 (92.59%)	2 (7.41%)	0 (0.00%)		
Fits better into the work of anesthesiologists	22 (81.48%)	4 (14.81%)	1 (3.70%)		
More accurately records performed procedures	23 (85.19%)	4 (14.81%)	0 (0.00%)		
Enables better collaboration within the anesthesia team	16 (59.26%)	7 (25.93%)	4 (14.81%)		
Records administered medications more accurately	22 (81.48%)	4 (14.81%)	1 (3.70%)		
Provides more time to work on the patient	22 (81.48%)	4 (14.81%)	1 (3.70%)		
Safer for the patient	18 (66.67%)	9 (33.33%)	0 (0.00%)		
Ensures better long-term storage and preservation of records	25 (92.59%)	1 (3.70%)	1 (3.70%)		
Personally preferred form of anesthesia record keeping	24 (88.89%)	2 (7.41%)	1 (3.70%)		
Easier for subsequent analysis of anesthesia and events	13 (48.15%)	7 (25.93%)	7 (25.93%)		
More flexible for future improvements and adjustments	24 (88.89%)	3 (11.11%)	0 (0.00%)		
	I completely agree	I partially agree	I am indifferent	I partially disagree	I completely disagree
Diane was intuitive to use	10 (38.46%)	8 (30.77%)	0 (0.00%)	5 (19.23%)	3 (11.54%)
Diane is easy to use without prior training	6 (23.08%)	9 (34.62%)	0 (0.00%)	8 (30.77%)	3 (11.54%)
I am familiar with all the features of Diane	5 (18.52%)	12 (44.44%)	0 (0.00%)	9 (33.33%)	1 (3.70%)
I don’t waste time searching for the necessary functions in the Diane system	4 (16.00%)	11 (44.00%)	0 (0.00%)	9 (36.00%)	1 (4.00%)
If I can’t find a function in Diane at first, I know where to look for it	9 (34.62%)	9 (34.62%)	0 (0.00%)	6 (23.08%)	2 (7.69%)
I have received sufficient training to use the Diane system	9 (36.00%)	9 (36.00%)	0 (0.00%)	4 (16.00%)	3 (12.00%)
I have access to fast and efficient technical support for Diane	9 (39.13%)	7 (30.43%)	0 (0.00%)	5 (21.74%)	2 (8.70%)
The Diane system is continuously improving according to user needs	13 (52.00%)	7 (28.00%)	0 (0.00%)	2 (8.00%)	3 (12.00%)
It takes more time to fully get used to working with Diane than I expected	2 (9.52%)	4 (19.05%)	0 (0.00%)	6 (28.57%)	9 (42.86%)
Getting used to work with Diane negatively affected my ability to provide patient care	1 (3.85%)	2 (7.69%)	0 (0.00%)	4 (15.38%)	19 (73.08%)
	Yes	Some	No		
Have you watched the educational videos for Diane?	5 (18.52%)	13 (48.15%)	9 (33.33%)		
	Always	Often	Sometimes	Rarely	Never
Diane guides me through its menus to complete my records more completely	3 (11.11%)	11 (40.74%)	8 (29.63%)	2 (7.41%)	3 (11.11%)
I rely on Diane to remind me of the necessary steps to provide better anesthesia	2 (7.41%)	6 (22.22%)	7 (25.93%)	9 (33.33%)	3 (11.11%)
Diane helps me reduce the possibility of documentation and treatment errors	4 (14.81%)	10 (37.04%)	6 (22.22%)	4 (14.81%)	3 (11.11%)
I use the automatic tidal volume calculator to set up mechanical ventilation	4 (14.81%)	2 (7.41%)	6 (22.22%)	5 (18.52%)	10 (37.04%)
I use the SmofKabiven Extra Nitrogen calculator	4 (14.81%)	6 (22.22%)	4 (14.81%)	4 (14.81%)	9 (33.33%)
I use the guidelines summary in Diane	2 (7.41%)	3 (11.11%)	7 (25.93%)	9 (33.33%)	6 (22.22%)
The automatic calculations in Diane facilitate more accurate and safer drug dosing	9 (33.33%)	10 (37.04%)	4 (14.81%)	3 (11.11%)	1 (3.70%)

## Data Availability

The data presented in this study are available on request from the corresponding author due to institutional and GDPR regulations.

## Abbreviations

The following abbreviations are used in this manuscript:

AIMS	Anesthesia Information Management System
PDMS	Patient Data Management System
